# QiangGuYin Modulates the OPG/RANKL/RANK Pathway by Increasing Secretin Levels during Treatment of Primary Type I Osteoporosis

**DOI:** 10.1155/2021/7114139

**Published:** 2021-10-31

**Authors:** Xuefei Li, Longkang Cui, Wenhua Chen, Yuan Fang, Gaobo Shen, Zhen Li, Bingbing Zhang, Lianguo Wu

**Affiliations:** ^1^Second Clinical Medical College, Zhejiang Chinese Medical University, Hangzhou 310053, China; ^2^The Second Affiliated Hospital, Zhejiang Chinese Medical University, Hangzhou 310005, China; ^3^School of Pharmaceutical Sciences, Zhejiang Chinese Medical University, Hangzhou 310053, China

## Abstract

QiangGuYin (QGY) is a common Traditional Chinese medicine prescription for the treatment of osteoporosis. Previous clinical studies have found that QGY effectively improves bone mineral density (BMD) in postmenopausal women, but its underlying mechanism remains unclear. The osteoprotegerin (OPG)/receptor activator of nuclear factor kappa B ligand (RANKL)/receptor activator of nuclear factor kappa B (RANK) pathway is a classic pathway involved in osteoporosis. Secretin levels are a serum marker of osteoporosis, but their effect on the OPG/RANKL/RANK pathway has not been reported. Hence, we investigated the relationship between the OPG/RANKL/RANK pathway and secretin and further revealed the mechanism underlying the effect of QGY in the treatment of osteoporosis. Mice were divided into secretin knockdown, secretin overexpression, and corresponding control groups. Micro-computed tomography was used to detect BMD in different groups, and the results show that QGY significantly improved BMD in mice of the secretin knockdown group. To further verify this, the serum levels of OPG, RANKL, RANK, and secretin were measured by enzyme-linked immunosorbent assays, and femur levels of OPG, RANKL, RANK, and secretin were evaluated by real-time quantitative PCR and western blotting. The results show that the expression of OPG was inhibited and that of RANKL and RANK was increased in mice from the secretin knockdown group, whereas the expression of OPG was upregulated and that of RANKL and RANK was downregulated after QGY intervention. Therefore, QGY inhibited bone resorption by promoting the expression of secretin and modulating the OPG/RANKL/RANK pathway. In addition to the effect of QGY, we also revealed the general regulatory effect of secretin on the OPG/RANKL/RANK pathway. We conclude that QGY modulates the OPG/RANKL/RANK pathway by increasing secretin levels during treatment of primary type I osteoporosis. This work provides a theoretical basis for the clinical use of QGY in the treatment of osteoporosis.

## 1. Introduction

Osteoporosis has become a public health issue worldwide and is considered a major health problem, second only to coronary heart disease, by the World Health Organization [1]. Osteoporosis is a skeletal disease characterized by an imbalance between bone resorption and bone formation, which leads to low bone mineral density (BMD) and microarchitectural deterioration of bone tissue, accompanied by a consequently higher bone fragility and susceptibility to fracture [2–4]. Furthermore, it was estimated that the prevalence of osteoporosis in the Chinese population (greater than 50 years of age) will increase from 5.04% (1990) to 7.46% (2050) in men and from 26.28% (1990) to 39.19% (2050) in women [5]. The drugs used to treat osteoporosis include vitamin D analogs, calcitonin, bisphosphonates, estrogen, selective estrogen receptor modulators, and parathyroid hormone [6, 7]. In general, drug intervention for osteoporosis may be divided into two categories, bone anabolic agents and antibone resorption agents. Unfortunately, there are several side effects, such as fever, vomiting, atypical femoral fractures, and jaw necrosis, associated with these drugs, thereby compromising quality of life [8]. Traditional Chinese medicine (TCM) has a long history in the treatment of osteoporosis [9]. Developing a new antiosteoporosis TCM prescription might provide a novel strategy for the treatment of osteoporosis.

It is well known that osteoporosis is closely associated with diseases of the digestive system [10–13]. Secretin, a hormone, is secreted by duodenal S cells [14] and plays an important role in the pathological processes of digestive system diseases and osteoporosis. Previous studies have revealed that secretin is a serum protein marker of type I osteoporosis in postmenopausal women [15, 16]. Structurally, secretin is closely associated with calcitonin, a hormone that inhibits osteoclast function [10, 13, 17]. However, secretin was also known to decrease gastric H^+^ secretion in the duodenum [15], thus inhibiting calcium absorption, resulting in low bone mass [18]. However, the mechanism of action of secretin in osteoporosis remains unclear. Meanwhile, it has been reported that the osteoprotegerin (OPG)/receptor activator of nuclear factor kappa B ligand (RANKL)/receptor activator of nuclear factor kappa B (RANK) signaling pathway is a major regulator of osteoporosis [4, 19]. Therefore, we speculated that secretin might play a crucial role in regulating the OPG/RANKL/RANK signaling pathway in bone remodeling in primary type I osteoporosis.

QiangGuYin (QGY) is a TCM prescription that has been widely used in the prevention and treatment of osteoporosis clinically, and its effectiveness and safety have been previously established [1, 3]. In TCM theory, QGY has been reported to possess tonifying “Qi” and warming meridians. Moreover, it was reported that QGY relieves clinical symptoms of pain, deformation of the spine, and fractures by improving BMD and reducing fracture risk. QGY consists of 10 herbal drugs, *Cervi Cornu Degelatinatum*, *Hedysarum multijugum* Maxim., *Drynariae Rhizoma*, *Eucommiae Cortex*, *Chuanxiong Rhizoma*, *Spatholobus suberectus* Dunn., *Cinnamomi Cortex*, *Radix Angelicae biseratae*, *Gentiana macrophylla Pall*, and *Saposhnikoviae Radix*. *Cervi Cornu Degelatinatum*, *Hedysarum multijugum* Maxim., and *Drynariae Rhizoma* have long been used in TCM for strengthening bones and are listed as the gentlemen's medicines of QGY. Among them, polypeptides from *Cervi Cornu Degelatinatum* have been shown to prevent bone loss and promote the multiplication of osteoblast-like cells [20]. *Hedysarum multijugum* Maxim. [21] increases bone mineral density (BMD) content by improving the level of OPG and reducing the level of RANKL in ovariectomy-induced mice. *Drynariae Rhizoma* enhances the strength and hardness of bone [22]. Meanwhile, our previous studies reported that *Drynaria* flavonoids improve osteoporosis by promoting the expression of OPG and inhibiting RANKL and RANK in the sera of osteoporosis model rats [23]. Therefore, it is important to elucidate the relationship among the QGY prescription, osteoporosis, secretin, and the OPG/RANKL/RANK pathway. We hypothesized that QGY regulates OPG/RANKL/RANK pathway by improving the level of secretin to treat the primary type I osteoporosis. The purpose of this study was to investigate the therapeutic effects of QGY on bone loss and its possible mechanisms. In this study, we analyzed the mRNA and protein levels of secretin, OPG, RANKL, and RANK by enzyme-linked immunosorbent assays (ELISA), western blotting, and real-time quantitative PCR (RT-PCR). Moreover, we confirmed that the release of secretin modulated the OPG/RANKL/RANK pathway using secretin knockdown and secretin overexpression models. We believe that the results of this study may help develop an effective treatment for osteoporosis.

## 2. Materials and Methods

### 2.1. Preparation of QGY

QGY is composed of 10 herbs, including *Cervi Cornu Degelatinatum* (20 g), *Hedysarum multijugum* Maxim. (30 g), *Drynariae Rhizoma* (20 g), *Eucommiae Cortex* (15 g), *Chuanxiong Rhizoma* (30 g), *Spatholobus suberectus* Dunn. (25 g), *Cinnamomi Cortex* (10 g), *Radix Angelicae biseratae* (15 g), *Gentiana macrophylla Pall* (15 g), and *Saposhnikoviae Radix* (15 g). All herbs were purchased from the Second Affiliated Hospital of Zhejiang Chinese Medicine University (Zhejiang Province, China) and can be found in the Pharmacopeia of China (2020 edition, Chinese Medical Science and Technology Press). According to the TCM decoction method, all herbs were mixed and boiled for 1.5 h, and the process was repeated three times. The decoction was concentrated to obtain a 1.5 g/mL concentration of crude drug and preserved at 4°C.

### 2.2. Adeno-Associated Virus (AAV) Construction and Injection

Mice were injected with AAV vectors only once after 1 week of feeding, which mediated the efficient transduction of bone tissue at 5 × 10^10^ vector genomes/g body weight, followed by QGY administration. In this study, AAVs were resuspended in phosphate-buffered saline (PBS) and used. AAV carrying the secretin-targeting shRNA or scramble control shRNA was purchased from Genomeditech (Shanghai, China). The shRNA sequence was as follows: secretin AAV, 5′-AAAAGGACCAAGGTCTGCTGTTGATTTCGATCAACAGCAGACCTTGGTCC-3′.

### 2.3. Animal Grouping and QGY Administration

Forty-two healthy C57BL/6 mice (specific-pathogen-free (SPF) level, 6 months of age, female) were acquired from Peking Weitong Lihua Experimental Animal Co., Ltd. All experimental procedures strictly adhered to the Institutional Animal Care and Use Committee (IACUC) of the Zhejiang Chinese Medical University and were approved by the Committee on the Ethics of Animal Experiments of Zhejiang Chinese Medical University (11223). Animals were raised in an SPF environment for 1 week prior to the start of the study and were kept in standard environmental conditions of temperature (26 ± 2°C), humidity (70%), and light (12 h/12 h light/dark). Adeno-associated virus (AAV) was injected into the caudal vein to establish mouse models of secretin knockdown and secretin overexpression [24]. The DNA sequence for secretin overexpression was combined with the AAV vector containing a green fluorescent protein (GFP) sequence, which was named the secretin overexpression group. Similarly, an AAV-secretin knockdown mouse model was developed by injecting AAV carrying the secretin-targeting shRNA. The same AAV vector containing only GFP was used as a control and named the secretin overexpression control or secretin knockdown control groups. After 1 week of adaptive feeding, the C57BL/6 mice were randomly distributed into seven groups as follows: control group (Con), secretin overexpression group (Sec-over), secretin overexpression control group (Sec-over-Con), secretin overexpression + QGY group (Sec-over + Q), secretin knockdown group (Sec-kno), secretin knockdown control group (Sec-kno-Con), and secretin knockdown + QGY group (Sec-kno + Q). After tail vein injection of AAV, the mice were adaptively fed for 3 weeks and then divided into the relevant groups. Next, QGY was given by gavage for 8 weeks. The QGY treatment group was perfused with QGY at a dose of 350 mg/10 g/d, whereas the control groups were orally administered normal saline. Peripheral blood was collected from the carotid artery, and bilateral femurs were cryopreserved in liquid nitrogen for future use.

### 2.4. Micro-Computed Tomography (CT) Scan

The left femurs were conserved, as previously mentioned. The bone mass and trabecular microarchitecture from the middle femur and distal metaphysis were scanned using a high-resolution (12 *μ*m) micro-CT system (micro-CT 80, Scanco Medical, AG, Switzerland). The analyzed parameters included BMD, trabecular thickness (Tb. Th), trabecular number (Tb. N), bone volume/tissue volume (BV/TV), and trabecular separation (Tb. Sp), which were auto-calculated using a computer.

### 2.5. ELISA

Serum was obtained from peripheral blood specimens using standard methods and stored at −80°C until analysis. The levels of secretin, OPG, RANKL, and RANK in serum were tested using ELISA kits (Jingmei Biological Company, China), according to the manufacturer's instructions. Absorbance was measured using a SpectraMax Plus 384 (Molecular Devices, USA) with a test wavelength of 450 nm.

### 2.6. Western Blot Analysis

The frozen comminuted bone tissue specimens were minced and homogenized using radioimmunoprecipitation assay buffer (Beyotime Institute of Biotechnology, Jiangsu, China). After centrifugation at 5000 rpm at 4°C for 10 min, the supernatant was collected and stored at −80°C. The total protein concentration was determined using a BCA protein assay kit (Thermo, USA). Equal amounts of protein (20 *μ*g) were electrophoresed using 10% SDS-polyacrylamide gel electrophoresis (Merck, Germany) gels and transferred onto a 0.45 *μ*m polyvinylidene fluoride (PVDF) membrane (Millipore, USA). Furthermore, the PVDF membranes were blocked with 5% skim milk in tris-buffered saline (Merck, Germany) for 2 h and then incubated with primary antibodies, including those against secretin (1 : 1000, PA5-75673, Invitrogen, USA), OPG (1 : 1000, ab227387, Abcam, UK), RANK (1 : 1000, ab200369, Abcam, UK), and RANKL (1 : 1000, ab65024, Abcam, UK). The membrane was washed with PBS and incubated with the secondary horseradish peroxidase-conjugated goat anti-mouse/rabbit antibodies (1 : 2000, Abcam, UK) for 0.5 h at room temperature. After incubation, protein bands were quantified using ImageJ 1.52v software.

### 2.7. RT-PCR Analysis

The mRNA levels of secretin, OPG, RANKL, and RANK were determined by RT-PCR. Total RNA was separated from femurs using TRIzol Reagent (15596018, Thermo, USA) according to the manufacturer's protocol, and then, 1.0 *μ*g of total RNA was reverse transcribed into cDNA using the PrimeScript RT reagent kit (RR037B, Takara, Japan). PCR amplification was performed and normalized using TB Green® Premix Ex Taq™ (RR420B, Takara, Japan) and a 7500 Fast Real-time PCR instrument (Applied Biosystems, Foster City, USA). Relative mRNA levels were calculated using the 2^−ΔΔCt^ method [25]. The following primers were used for PCR: *OPG*, forward 5′-ACCCAGAAACTGGTCATCAGC-3′, reverse 5′-CTGCAATACACACACTCATCACT-3′; *RANK*: forward 5′-GGACGGTGTTGCAGCAGAT-3′, reverse 5′-GCAGTCTGAGTTCCAGTGGTA-3′; *RANKL*: forward 5′-CAGCATCGCTCTGTTCCTGTA-3′, reverse 5′-CTGCGTTTTCATGGAGTCTCA-3′; *β-actin*: forward 5′-GGCTGTATTCCCCTCCATCG-3′, reverse 5′-CCAGTTGGTAACAATGCCATGT-3′.

### 2.8. Statistical Analysis

All statistical analyses were conducted using SPSS 22.0 and GraphPad 8.0 Prism software. All data are expressed as the mean ± SD, and one-way analysis of variance was used to compare multiple groups. Statistical significance was set at *P* < 0.05.

## 3. Results

### 3.1. QGY Enhances BMD and Improves Bone Microstructure

**Figure 1:**
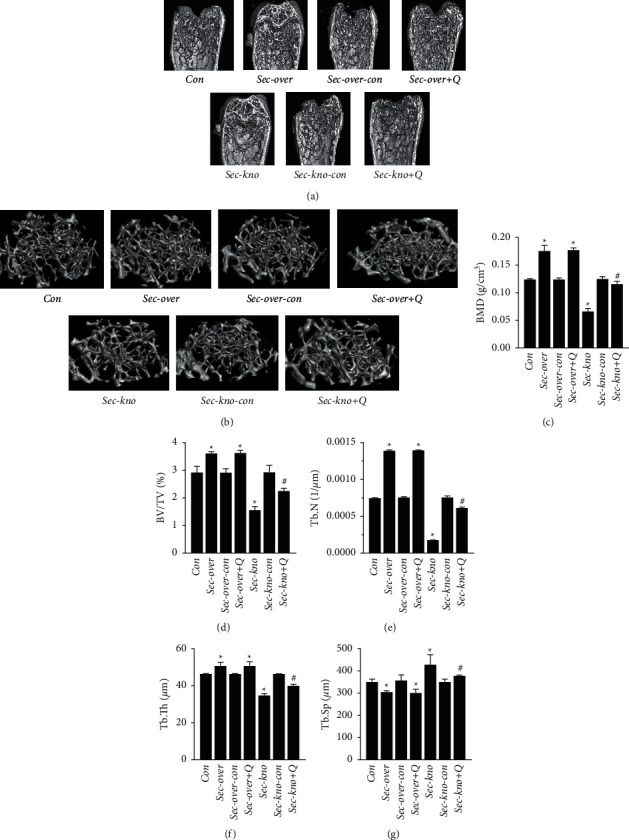
Representative 3D reconstruction images of bone microarchitecture of the coronal plane of bone of micro-CT (a) and distal femur micro-CT scans (b). Effect of QiangGuYin (QGY) treatment on bone parameters, including (c) bone mineral density (BMD), (d) bone volume/tissue volume (BV/TV), (e) trabecular number (Tb. N), (f) trabecular thickness (Tb. Th), and (g) trabecular separation (Tb. Sp) in the distal femurs of mice. Data are expressed as the mean ± SD (*n* = 6). ^*∗*^*P* < 0.05 vs. the Con group; ^#^*P* < 0.05 vs. the Sec-kno group. Groups are as follows: control group (Con), secretin overexpression group (Sec-over), secretin overexpression control group (Sec-over-Con), secretin overexpression + QGY group (Sec-over + Q), secretin knockdown group (Sec-kno), secretin knockdown control group (Sec-kno-Con), and secretin knockdown + QGY group (Sec-kno + Q).

BMD and bone structural properties have been widely used to evaluate the severity of osteoporosis. Our previous clinical studies have found that QGY significantly improves BMD in patients with osteoporosis and that secretin is a specific protein for osteoporosis. Therefore, we speculated that QGY might reduce bone loss by affecting the expression of secretin [1, 16]. To test this hypothesis, we examined the BMD and bone structural properties in Sec-over and Sec-kno mice. Micro-CT analysis of the femoral shaft revealed that BMD was decreased in the Sec-kno group and increased in the Sec-over group, as compared to that in the control groups. Importantly, BMD was increased in the Sec-kno group after QGY intervention (Figure 1). Furthermore, the morphological parameters of bone tissue indicated that the low expression of secretin resulted in a remarkable decrease in BV/TV and Tb. Th and Tb. N and an increase in Tb. Sp. In addition, morphological changes in trabecular bone suggested improvements after treatment with QGY. The results indicated that QGY increased BMD and improved bone microstructure in the Sec-kno group of mice.

### 3.2. QGY Increases Expression of Secretin in Serum

**Figure 2:**
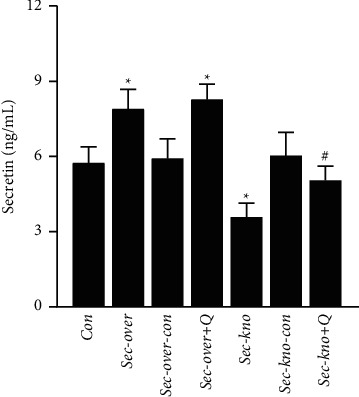
Effect of QiangGuYin (QGY) treatment on the expression of secretin in mice from different groups. Data are expressed as the mean ± SD (*n* = 6). ^*∗*^*P* < 0.05 vs. the Con group; ^#^*P* < 0.05 vs. the Sec-kno group. Groups are as follows: control group (Con), secretin overexpression group (Sec-over), secretin overexpression control group (Sec-over-Con), secretin overexpression + QGY group (Sec-over + Q), secretin knockdown group (Sec-kno), secretin knockdown control group (Sec-kno-Con), and secretin knockdown + QGY group (Sec-kno + Q).

We initially developed two animal models, Sec-kno and Sec-over, to explore the specific relationship between QGY and secretin. The expression of secretin in mice from the Sec-over and Sec-kno groups showed significant changes (^*∗*^*P* < 0.05) compared with that in mice from the Con group. Secretin levels were found to be significantly higher after QGY treatment in the Sec-over group, as compared to that before intervention. It was, therefore, verified that QGY could promote the expression of secretin (Figure 2).

### 3.3. QGY and Secretin Have Positive Regulatory Effects on the OPG/RANKL/RANK Pathway in Serum

**Figure 3:**
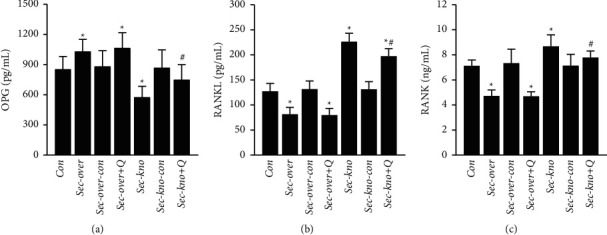
Effect of QiangGuYin (QGY) treatment and secretin expression on the expression levels of RANKL, RANK, and OPG in mouse serum. (a) OPG; (b) RANKL; (c) RANK. Data are expressed as the mean ± SD (*n* = 6). ^*∗*^*P* < 0.05 vs. the Con group; ^#^*P* < 0.05 vs. the Sec-kno group. Groups were as follows: control group (Con), secretin overexpression group (Sec-over), secretin overexpression control group (Sec-over-Con), secretin overexpression + QGY group (Sec-over + Q), secretin knockdown group (Sec-kno), secretin knockdown control group (Sec-kno-Con), and secretin knockdown + QGY group (Sec-kno + Q).

The OPG/RANKL/RANK pathway is a classic pathway involved in osteoporosis. To explore whether QGY and secretin were related to the OPG/RANKL/RANK signaling pathway, we monitored the expression of OPG, RANKL, and RANK in mouse serum from different treatment groups (by ELISA) after the oral administration of QGY. The results revealed that OPG was increased in mice of the Sec-over group and decreased in mice of the Sec-kno group (^*∗*^*P* < 0.05), as compared to that in the Con group, as shown in Figure 3(a). The expression of OPG was increased after the oral administration of QGY in the Sec-kno + Q group compared to that in the Sec-kno group (^#^*P* < 0.05). Simultaneously, the expression of RANKL and RANK decreased in the Sec-over group and increased in the Sec-kno group as compared to levels in the Con group. The expression of RANKL and RANK was significantly decreased after treatment with QGY as compared to that in mice of the Sec-kno group (Figures 3(b) and 3(c)). The results verified that QGY could regulate the OPG/RANKL/RANK pathway by affecting the expression of secretin in blood.

### 3.4. QGY Regulates the Expression of Secretin, OPG, RANKL, and RANK in Femur Tissue

**Figure 4:**
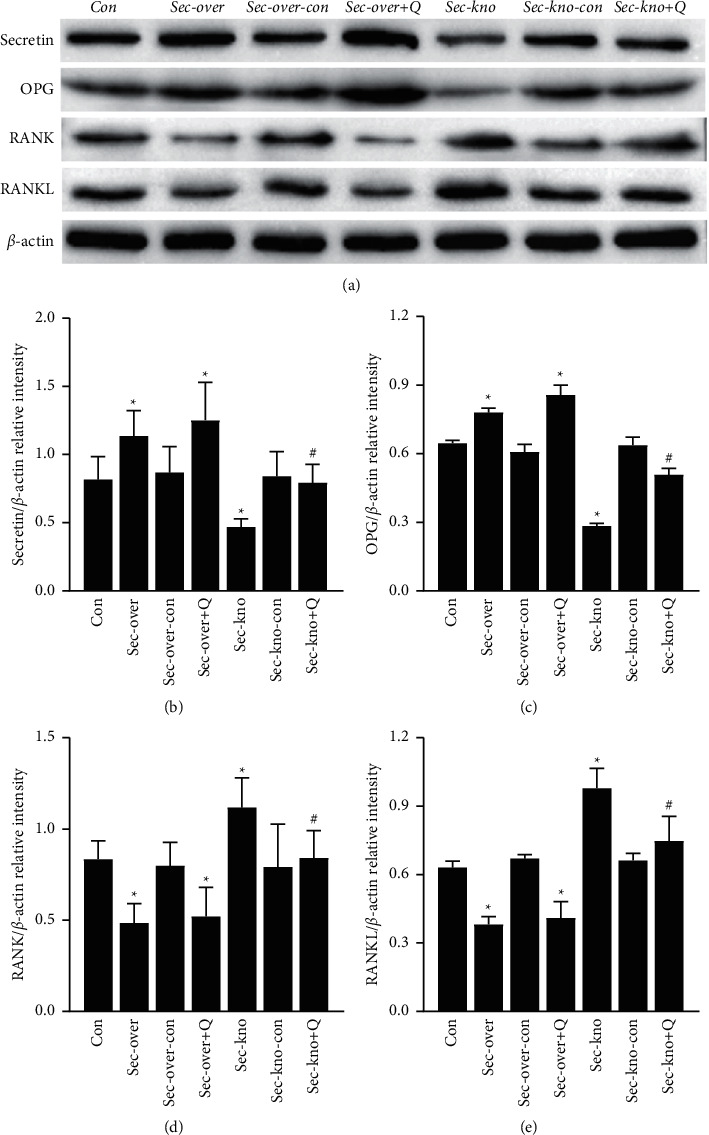
Effect of QiangGuYin (QGY) treatment on the protein expression of secretin, OPG, RANK, and RANKL in the femur tissue. (a–e) The protein expression of secretin and OPG was decreased, whereas that of RANK and RANKL was increased, after secretin knockdown. The expression of secretin and OPG was increased, whereas that of RANK and RANKL was significantly decreased in the Sec-kno + Q group, as compared to that in the Sec-kno group. Data are expressed as the mean ± SD (*n* = 3). ^*∗*^*P* < 0.05 vs. the Con group; ^#^*P* < 0.05 vs. the Sec-kno group. Groups were as follows: control group (Con), secretin overexpression group (Sec-over), secretin overexpression control group (Sec-over-Con), secretin overexpression + QGY group (Sec-over + Q), secretin knockdown group (Sec-kno), secretin knockdown control group (Sec-kno-Con), and secretin knockdown + QGY group (Sec-kno + Q).

**Figure 5:**
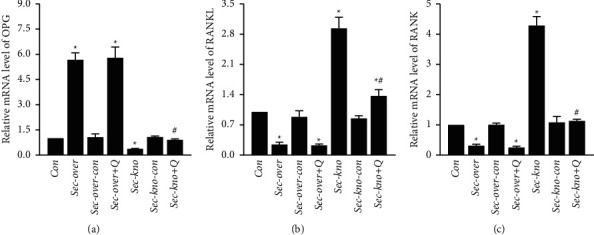
Effect of QiangGuYin (QGY) treatment on the mRNA expression of OPG, RANK, and RANKL in the femur tissue. (a–c) The mRNA expression of *OPG* was decreased, whereas that of *RANK* and *RANKL* was increased, after secretin knockdown. The mRNA expression of OPG was increased, whereas that of RANK and RANKL was significantly decreased, in the Sec-kno + Q group, compared to levels in the Sec-kno group. Data are expressed as the mean ± SD (*n* = 6). ^*∗*^*P* < 0.05 vs. the Con group; ^#^*P* < 0.05 vs. the Sec-kno group. Groups were as follows: control group (Con), secretin overexpression group (Sec-over), secretin overexpression control group (Sec-over-Con), secretin overexpression + QGY group (Sec-over + Q), secretin knockdown group (Sec-kno), secretin knockdown control group (Sec-kno-Con), and secretin knockdown + QGY group (Sec-kno + Q).

It is well known that OPG, RANKL, and RANK constitute a system that regulates bone remodeling. To further explore the potential mechanisms through which QGY exerts effect on osteoporosis, the expression of secretin, OPG, RANKL, and RANK in femur tissue was studied using western blotting and PCR. The western blot results are shown in Figure 4; secretin and OPG protein expression was downregulated, whereas that of RANKL and RANK was upregulated, in femur tissue from mice in the Sec-kno group. Meanwhile, the expression of secretin and OPG was increased and that of RANK and RANKL was decreased after treatment with QGY. PCR results are shown in Figure 5, and a similar trend was observed.

## 4. Discussion

Osteoporosis is a degenerative bone disease related to aging [7]. It is well known that an imbalance between bone resorption (driven by osteoclasts) and bone formation (driven by osteoblasts) results in decreased bone mass [5, 26]. Therefore, studying the mechanism of osteoblasts and osteoclasts could help in designing therapeutic strategies to effectively prevent a reduction in bone mass. Based on current knowledge, the OPG/RANL/RANK signaling pathway is the main regulator of osteoporosis and modulates osteoclast differentiation, induction, activation, and maintenance [19, 27]. RANKL binds to RANK on the surface of osteoclasts to form a RANKL–RANK complex, which stimulates the activation, formation, and differentiation of osteoclasts [28, 29]. OPG inhibits bone resorption by preventing the binding of RANKL to the RANK receptor by blocking the RANKL binding site [30, 31]. It has been reported that increased levels of RANKL/OPG directly affect osteoclasts [32]. In addition, digestive system diseases, especially inflammatory bowel disease, are significantly associated with an increased risk of osteoporosis [33]. The mechanism leading to low BMD and increased fracture risk caused by inflammatory bowel disease is closely related to OPG/RANKL/RANK pathway. In several studies, it has been shown that inflammatory bowel disease results in increased concentrations of proinflammatory cytokines, including tumor necrosis factor alpha (TNF-*α*), interleukin (IL)-1*β*, IL-6, and IL-17 [34]. It is well known that cytokines such as TNF-*α*, IL-1*β*, and IL-6 increase the production of receptor activator of nuclear factor kappa B ligand (RANKL) by preosteoblasts, thereby promoting osteoclastogenesis. These cytokines bind to their respective receptors, which causes increased activation of the *p*38 mitogen-activated protein kinase (MAPK), nuclear factor kappa B (NF-*κ*B), and c-Jun N-terminal kinase (JNK) pathways, resulting in excessive bone loss and reduced BMD [35]. On the other hand, digestive system diseases lead to malabsorption of trace elements such as calcium, vitamin C, and vitamin D [36]. Studies suggest that insufficient calcium intake leads to excessive parathyroid hormone production and decreased BMD. Vitamin C is absorbed in the small intestine, and a higher consumption of this vitamin decreases the risk of osteoporosis by 33% [37]. Vitamin D acts directly and indirectly on the bone, regulates calcium absorption in the gastrointestinal tract, determines the proper serum calcium level, influences osteoblasts mineralization and differentiation, and decreases the synthesis of parathormone and the reabsorption of phosphates from bones [38]. Secretin protein was the first animal hormone discovered by Bayliss and Starling in 1902 [39]. The cells producing secretin were named “S” cells and are mainly distributed in the duodenal mucosa, with a small number in the jejunum, ileum, and gastric antrum. It was found that secretin has similar functions with PTH and calcitonin, and there is an important relationship between secretin and RANKL of the OPG/RANKL/RANK pathway of bone metabolism, as shown by the string protein-protein interaction network analysis tool. The OPG/RANKL/RANK pathway and its downstream factors are relatively well studied, but it is not clear whether there are influencing factors or proteins upstream of this pathway. Based on our experimental results, we propose a superior subordinate relationship, the secretin/OPG/RANKL/RANK pathway. These results also show the close relationship between digestive system diseases and osteoporosis.

Osteoporosis has always been a research hotspot in orthopedics and other related fields. There are many methods to obtain animal models of osteoporosis, such as surgical induction [40] (removal of bilateral ovaries/testes), drug induction [41] (adrenocortical hormone and other drugs), loss induction, diet induction [42] (low calcium diet), gene knockout [43] (knockout of the OPG gene), and spontaneous degeneration. The molding time is generally about 2 months. In our previous study, we found that secretin is a specific serum protein marker for patients with type I osteoporosis [2]. Therefore, we used the AAV-mediated knockdown of the secretin gene to obtain an osteoporosis mouse model. After 3 weeks, we evaluated whether the model was successful by determining BMD, an important index to evaluate bone strength [44]. According to the micro-CT analysis of femoral shafts, it was revealed that BMD was decreased in mice from the Sec-kno group and increased in mice from the Sec-over group as compared to that in mice from Con groups. Moreover, morphological parameters of bone tissue indicated that the low expression of secretin resulted in a significant decrease in BV/TV, Tb. Th, and Tb. N and an increase in Tb. Sp. This experiment showed that our method of obtaining an osteoporosis model with secretin knockdown was successful, and the required time was much shorter compared with previous methods. The morphological changes of trabecular bone were also improved after treatment with QGY. It was found that QGY improved BMD and bone microstructure in Sec-kno-group mice. Thus, it was important to elucidate the mechanistic details of the QGY function that led to an effective improvement in BMD in mice, as well as the role that secretin played in this process. ELISA and western blot assays indicated that QGY had a positive effect on bone loss in mice from the Sec-kno group. The results also indicated that BMD in mice from the Sec-kno group was significantly decreased but that the level of BMD was significantly improved after QGY treatment. It has been suggested that QGY has a positive effect on BMD by increasing the level of secretin. Moreover, RANKL, RANK, and OPG constitute a three-in-one system regulating bone resorption and bone formation [45]. In our experiments, we detected the protein expression of RANKL, RANK, and OPG in both serum and bone tissue by ELISA and western blotting, as well as the mRNA levels of RANKL, RANK, and OPG in bone tissue by RT-qPCR. The results demonstrated that knockdown of secretin inhibited OPG expression and promoted RANKL and RANK expression. After the oral administration of QGY, the expression of RANKL and RANK was inhibited, whereas OPG expression was upregulated. The experimental results support our hypothesis and revealed the antiosteoporosis mechanism of QGY. Our previous clinical trials found that QGY significantly improves BMD and reduces clinical symptoms such as low back pain in patients with osteoporosis but failed to reveal the underlying mechanism [1]. The present work used the new modeling method of secretin knockdown, which shows that QGY modulates the OPG/RANKL/RANK pathway by increasing secretin levels during treatment of primary type I osteoporosis.

Recent studies have shown that QGY is widely used in the prevention and treatment of osteoporosis [1]. Pharmacological studies have suggested that the effective components of natural Chinese medicine can attenuate the imbalance in bone resorption and reduce bone microstructural degradation [9, 46, 47]. Interestingly, the active ingredients in the prescription of QGY play an important role in preventing low bone mass [7, 48–52]. Polysaccharides and total flavonoids were determined to be the active ingredients of *Hedysarum multijugum* Maxim., which could reduce bone resorption and inhibit osteoclasts by increasing OPG levels and reducing RANKL and RANK levels considerably, thereby lowering the ratio of RANKL/OPG. Moreover, polysaccharides effectively inhibit osteoclastogenesis by reducing tumor necrosis factor- (TNF-) *α* [7, 46]. Antler polypeptide is the active ingredient of *Colla Cornus Cervi*, which increases osteoblast differentiation and inhibits TNF-*α*-mediated inhibition of osteoclastogenesis via regulation of the NF-*κ*B/*p*65 pathway, as well as suppressing the NF-*κ*B signaling pathway in osteoclasts *in vitro* [48]. Flavonoids are the active ingredient of *Drynariae Rhizoma*, which decrease the levels of TNF-*α* and interleukin-6 (IL-6), involved in the induction of bone resorption, and suppress osteoclastogenesis by modulating the OPG/RANKL system in osteoblastic cells [48]. *Eucommiae Cortex*, rich in polyphenols such as phenolic acids, flavonoids, and lignans, is a kidney-tonifying herbal medicine that has been shown to improve bone histomorphometric parameters in lead-exposed rats. The possible mechanism underlying the effects of *Eucommia Cortex* extract is the promotion of bone formation by increasing the expression of OPG and the suppression of bone resorption by decreasing the level of RANKL in serum [22]. Gentiopicroside is the main active ingredient of *Gentiana macrophylla* Pall., which significantly suppresses RANKL-induced osteoclast formation in bone marrow mesenchymal stem cells (BMMs), with the possible mechanism being the inhibition of RANKL-induced activation of NF-*κ*B and Jun N-terminal kinase (JNK) signaling pathways in BMMs [50]. Previous studies have also found that *Saposhnikoviae divaricata* chromone extract reduces the levels of NF-*κ*B by suppressing *p*-*p*38, *p*-mitogen-activated protein kinase 1 (p-ERK), and p-JNK expression, which might be involved in the transcription of proinflammatory factors and the phosphorylation of the transcription factor NF-*κ*B. Moreover, it could suppress the production of IL-6, TNF-*α*, and IL-1*β* in tissues [52].

## 5. Conclusion

Our findings demonstrate that QGY modulates the OPG/RANKL/RANK pathway by increasing secretin levels during treatment of primary type I osteoporosis. In addition to the effect of QGY, we also revealed the general regulatory effect of secretin on the OPG/RANK/RANKL pathway, which has not been reported. Therefore, QGY might be a promising drug candidate for treating primary type I osteoporosis.

## Data Availability

The data are reflected in the manuscript; for more information, contact the corresponding author: Dr. Wu Lianguo, email: zcmu@vip.163.com.

## Abbreviations

QGY: QiangGuYin
BMD: Bone mineral density
TCM: Traditional Chinese medicine
AAV: Adeno-associated virus
OPG/RANK/RANKL: Osteoprotegerin/receptor activator of the nuclear factor kappa B (NF-*κ*B)/receptor activator of the NF-*κ*B ligand
ELISA: Enzyme-linked immunosorbent assay
ELISA: Enzyme-linked immunosorbent assay
RT-PCR: Real-time quantitative PCR
SPF: Specific-pathogen-free
GFP: Green fluorescent protein
Con: Control group
Sec-over: Secretin overexpression group
Sec-over-Con: Secretin overexpression control group
Sec-over + Q: Secretin overexpression + QGY group
Sec-kno: Secretin knockdown group
Sec-kno-Con: Secretin knockdown control group
Sec-kno + Q: Secretin knockdown + QGY group
PBS: Phosphate-buffered saline
Tb. Th: Trabecular thickness
Tb. N: Trabecular number
BV/TV: Bone volume/tissue volume
Tb. Sp: Trabecular separation
PVDF: Polyvinylidene fluoride
TNF-*α*: Tumor necrosis factor
IL-6: Interleukin-6
BMMs: Bone marrow mesenchymal stem cells
JNK: Jun N-terminal kinase.
